# Phylogenetic and Pathotypic Characterization of Newcastle Disease Viruses Circulating in South China and Transmission in Different Birds

**DOI:** 10.3389/fmicb.2016.00119

**Published:** 2016-02-09

**Authors:** Yinfeng Kang, Bin Xiang, Runyu Yuan, Xiaqiong Zhao, Minsha Feng, Pei Gao, Yanling Li, Yulian Li, Zhangyong Ning, Tao Ren

**Affiliations:** ^1^Key Laboratory of Animal Vaccine Development, College of Veterinary Medicine, South China Agricultural UniversityGuangzhou, China; ^2^Key Laboratory of Zoonosis Prevention and Control of Guangdong ProvinceGuangzhou, China; ^3^Key Laboratory for Repository and Application of Pathogenic Microbiology, Research Center for Pathogens Detection Technology of Emerging Infectious Diseases, Guangdong Provincial Center for Disease Control and PreventionGuangzhou, China

**Keywords:** newcastle disease virus (NDV), phylogenetic analysis, pathogenicity, transmission, South China

## Abstract

Although Newcastle disease virus (NDV) with high pathogenicity has frequently been isolated in poultry in China since 1948, the mode of its transmission among avian species remains largely unknown. Given that various wild bird species have been implicated as sources of transmission, in this study we genotypically and pathotypically characterized 23 NDV isolates collected from chickens, ducks, and pigeons in live bird markets (LBMs) in South China as part of an H7N9 surveillance program during December 2013–February 2014. To simulate the natural transmission of different kinds of animals in LBMs, we selected three representative NDVs—namely, GM, YF18, and GZ289—isolated from different birds to evaluate the pathogenicity and transmission of the indicated viruses in chickens, ducks, and pigeons. Furthermore, to investigate the replication and shedding of NDV in poultry, we inoculated the chickens, ducks, and pigeons with 10^6^ EID_50_ of each virus via intraocular and intranasal routes. Eight hour after infection, the naïve contact groups were housed with those inoculated with each of the viruses as a means to monitor contact transmission. Our results indicated that genetically diverse viruses circulate in LBMs in South China's Guangdong Province and that NDV from different birds have different tissue tropisms and host ranges when transmitted in different birds. We therefore propose the continuous epidemiological surveillance of LBMs to support the prevention of the spread of these viruses in different birds, especially chickens, and highlight the need for studies of the virus–host relationship.

## Introduction

Due to its high morbidity and mortality, Newcastle disease (ND) is considered to be the most significant and widespread infectious disease in commercial poultry—one that can cause severe economic losses in poultry, particularly chickens, and affect a range of other domestic species, including ducks and pigeons (Sinkovics and Horvath, 2000; OIE, 2012). In chickens, the clinical manifestation of NDV strains varies significantly due to the degree of strain virulence and host susceptibility (Alexander, 2000). NDV strains are categorized as highly virulent (velogenic), moderately virulent (mesogenic), or avirulent (lentogenic), all according to pathogenicity for chickens gauged by the intracerebral pathogenicity index (ICPI) and mean death time (MDT; OIE, 2012).

Historically, NDV strains have been divided into two major divisions—class I and class II—which contribute to the extensive genetic diversity among poultry worldwide, with class I being further divided into nine genotypes distributed worldwide in waterfowls and class II comprising 18 (I–XVIII) genotypes when the sequences are isolated over time (Ballagi-Pordány et al., 1996; Liu et al., 2003; Kim et al., 2007; Miller et al., 2010; Diel et al., 2012; OIE, 2012; Snoeck et al., 2013). With the exception of 9a5b, class I strains are avirulent in chickens and have historically been recovered from aquatic birds (Shengqing et al., 2002; Kim et al., 2007; Diel et al., 2012). Genotypes I, II, V, VI, VII, and IX of NDV have often been isolated in South China (e.g., Guangdong Province), although genotypes V, VI, and VII of class II strains are the predominant genotypes there and contain only virulent viruses (Jinding et al., 2005; Cai et al., 2011; Kang et al., 2014; Wang et al., 2015). In 1948, genotype IX emerged as a unique group including the first virulent NDV strains (F48E9) in South China, and members of the genotype are occasionally isolated from a wild variety of bird species (Liu et al., 2003). To date, many newly emergent strains isolated from a wide range of birds contribute to increasing the global burden of NDV and cause enormous losses in the poultry industry (Liu et al., 2003; Jinding et al., 2005; Kim et al., 2007; Kang et al., 2014). In 1981, genotype VI of NDV strains was first isolated from pigeons and was repeatedly isolated in China until 1985, when genotype VII became more epidemic and posed a constant threat to domestic poultry (Mase et al., 2002; Liu et al., 2003; Cai et al., 2011). Genotype VII of NDV strains have continued to circulate in poultry throughout South China and are now considered to be enzootic as they spread around the globe.

NDV has a wide range of hosts, as more than 250 bird species have been found to be susceptible by natural or experimental infections, although wild waterfowl and shorebirds are regarded to be the reservoir of the virus in nature (Kaleta and Baldauf, 1988). Chickens, pigeons, and ducks are most commonly susceptible to infection with the same NDV strain, though they exhibit different susceptibility (Erickson et al., 1980; Cattoli et al., 2011; Smietanka et al., 2014). As the most susceptible poultry, chickens are subject to high morbidity and mortality if infected with virulent NDV (Bogoyavlenskiy et al., 2005; Kang et al., 2014). Aquatic birds, including ducks, as natural reservoirs of NDV show nearly no obvious clinical symptoms when infected with NDV strains, even those virulent in chickens (Zhang et al., 2011; Kang et al., 2014). However, since the first large-scale outbreak of ND in geese in South China in 1997, duck-origin NDV strains have exhibited high virulence in waterfowl (Jinding et al., 2005; Zhang et al., 2011).

Some type 1 pigeon paramyxoviruses (PPMV-1) behave similarly to non-virulent viruses according to ICPI tests in 1-day-old chickens; whereas the strains are highly pathogenic to pigeons, the serial passaging of PPMV-1 in chickens results in increased virulence (Kommers et al., 2003; Dortmans et al., 2011). As a result, upon the natural transmission from pigeons to chickens, PPMV-1 strains may evolve into virulent viruses and induce major outbreaks (Dortmans et al., 2011). In China, the implementation of extensive vaccination procedures among commercial poultry farms and the culling of infected poultry have reduced the number of epizootic outbreaks of ND since the 1980s (Liu et al., 2003; Kang et al., 2014). However, genotypes V, VI, VII, and IX of NDV continue to circulate and frequently cause outbreaks in China (Cai et al., 2011; Qiu et al., 2011; Zhang et al., 2011; Kang et al., 2014; Wang et al., 2015). Nevertheless, very little information is available regarding the epidemiology, evolutionary trends, and transmission of the virus circulating in South China.

Previous studies have shown that PPMV-1 can be transmitted from infected pigeons to chickens placed in naïve contact (Alexander and Parsons, 1984; de Oliveira Torres Carrasco et al., 2008). At the same time, duck-origin NDV isolate can infect chickens and ducks and be transmitted to naïve contact chickens and ducks (Kang et al., 2014). However, little information is available regarding the pathogenesis and transmission of NDV among chickens, ducks, and pigeons. In this study, we therefore investigated the presence of NDV in chickens, ducks, and pigeons in live bird markets (LBMs) in South China and selected three viruses isolated from the different birds to better evaluate the pathogenicity and transmission of the NDV among various types of bird.

## Materials and methods

### Ethics statement

The present study was carried out in strict accordance with the recommendations in the Guide for the Care and Use of Laboratory Animals of the Ministry of Science and Technology of the People's Republic of China. All animal experiments were performed in animal biosafety level 3 facilities and were conducted under the guidance of South China Agricultural University's Institutional Animal Care and Use Committee and the Association for Assessment and Accreditation of Laboratory Animal Care International accredited facility. The protocol was reviewed and approved by the Committee on the Ethics of Animal Experiments of the animal biosafety level 3 Committee of South China Agricultural University, the approve ID is SCXK (Guangdong) 2013-0019.

### Virus isolation and biological characterization

Isolates were collected in LBMs in South China's Guangdong Province as part of an avian influenza (H7N9) surveillance program during December 2013–February 2014 by the Key Laboratory of Zoonosis Prevention and Control of Guangdong, China. Oropharyngeal and cloacal swab specimens were collected from commercial chickens, ducks, and pigeons from LBMs, also in Guangdong Province. A total of 214 swab samples were collected in 1.5 mL centrifuge tubes containing 1.0 mL transport medium (50% glycerol in phosphate-buffered saline [PBS]) with antibiotics (penicillin, 2000 U/mL; amphotericin B, 2000 U/mL; streptomycin, 2 mg/mL) and shipped to South China Agricultural University. The oropharyngeal and cloacal swab samples were inoculated in 10-days-old specific-pathogen-free (SPF) embryonated chicken eggs, as previously described (Kang et al., 2015). A hemagglutination (HA)-inhibition (HI) test was conducted using four HA units of the isolates and virus purification performed, as described by Office International Des Epizooties (OIE; OIE, 2012). A total of 23 NDV strains were isolated (Table 1). The MDT in 9-days-old SPF embryonated chicken eggs and ICPI tests in 1-day-old SPF chickens were performed according to OIE's (2012) standard procedure. Isolates were titrated in 10-days-old SPF-embryonated chicken eggs and stored at −80°C for further characterization. The evaluation of 50% egg infective doses (EID_50_) was calculated by using the Reed–Muench method (Reed and Muench, 1938).

**Table 1:** **Epidemiological, genetic, and biological characteristics description of 23 NDVs isolated from South China**.

**NDV isolate^a^**	**Host**	**Location (City)**	**Class**	**Genotype**	**MDT^b^(h)**	**ICPI^c^**	**F-protein cleavage site^d^**	**Pathotype**	**GenBank accession no.^e^**
Pigeon/Guangdong/GZ293/2014^*^	Pigeon	Guangzhou	Class II	VI	78	1.37	RRQKRF	Velogenic	KT381592
Chicken/Guangdong/GM5/2013 &^*^	Chicken	Gaoming	Class II	VII	70	1.73	RRQKRF	Velogenic	KT381593
Chicken/Guangdong/GM/2014 &^*^^f^	Chicken	Gaoming	Class II	VII	56	1.78	RRQKRF	Velogenic	DQ486859
Duck/Guangdong/YF21/2014 &^*^	Duck	Yunfu	Class II	VII	58	1.65	RRQKRF	Velogenic	KT381594
Pigeon/Guangdong/GM1/2014 &	Pigeon	Gaoming	Class II	VI	64	1.41	RRQKRF	Velogenic	KT381595
Pigeon/Guangdong/GM8/2013^*^	Pigeon	Gaoming	Class II	VI	87	1.29	KRQKRF	Velogenic	KT381596
Chicken/Guangdong/GZ295/2014	Chicken	Guangzhou	Class II	I	>120	0.5	RKQGRL	Lentogenic	KT381597
Duck/Guangdong/GM12/2014	Duck	Gaoming	Class II	I	>120	0.2	RKQGRL	Lentogenic	KT381598
Duck/Guangdong/GZ331/2014	Duck	Guangzhou	Class II	I	>120	0.2	RKQGHL	Lentogenic	KT381599
Duck/Guangdong/YF24/2014 &^*^	Duck	Yunfu	Class II	II	>120	0.2	GRQGRL	Lentogenic	KT381600
Pigeon/Guangdong/GZ287/2013 &	Pigeon	Guangzhou	Class II	VI	78	1.22	RRQKRF	Velogenic	KT381601
Pigeon/Guangdong/GZ288/2013 &	Pigeon	Guangzhou	Class II	VI	76	1.22	RRQKRF	Velogenic	KT381602
Pigeon/Guangdong/GZ290/2013	Pigeon	Guangzhou	Class II	VI	84	1.35	RRQKRF	Velogenic	KT381603
Pigeon/Guangdong/GZ292/2014	Pigeon	Guangzhou	Class II	VI	78	1.38	RRQKRF	Velogenic	KT381604
Duck/Guangdong/YF18/2014 &^*^	Duck	Yunfu	Class II	IX	42	1.83	RRQRRF	Velogenic	KT381605
Pigeon/Guangdong/GZ289/2014 &	Pigeon	Guangzhou	Class II	VI	58	1.72	RRQKRF	Velogenic	KT381606
Duck/Guangdong/YF821/2014 &^*^	Duck	Yunfu	Class I	3	>120	0	ERQERL	Lentogenic	KT381585
Duck/Guangdong/YF827/2014 &^*^	Duck	Yunfu	Class I	3	>120	0	ERQERL	Lentogenic	KT381586
Pigeon/Guangdong/YF1/2014	Pigeon	Yunfu	Class I	3	>120	0	ERQERL	Lentogenic	KT381587
Chicken/Guangdong/GM3/2013	Chicken	Gaoming	Class I	3	>120	0	ERQERL	Lentogenic	KT381588
Chicken/Guangdong/GM307/2014	Chicken	Gaoming	Class I	3	>120	0	ERQERL	Lentogenic	KT381589
Chicken/Guangdong/GZ316/2014	Chicken	Guangzhou	Class I	3	>120	0	ERQERL	Lentogenic	KT381590
Chicken/Guangdong/GZ379/2014	Chicken	Guangzhou	Class I	3	>120	0	ERQERL	Lentogenic	KT381591

a NDV was isolated from the oropharyngeal swab samples (

& ) or from both oropharyngeal and cloacal swab samples from the same bird (

* *). NDV isolates without any symbolic notation were isolated from cloacal swabs only*.

b *Mean death time in 10 d-old SPF embryonated chicken eggs (hours) (< 60, velogen; 60–90, mesogen; >90, lentogen)*.

c *Intracerebral pathogenicity index in day-old chickens (< 0.7, lentogen; 0.7–1.4, mesogen; 1.4–2.0, velogen)*.

d *Amino acids 112–117*.

e *The Gen Bank accession numbers provided are for the 1662-bp nucleotide sequence of the NDV-F gene open reading frame of South China NDV strains*.

f *Published by Wang et al. (2014)*.

### Animals

To test pathogenicity and reproduce the natural conditions of NDV transmission in chickens, ducks, and pigeons, we selected three strains representative of and circulating in South China: genotype VII Chicken/Guangdong/GM/2014 (GM), genotype IX Duck/Guangdong/YF18/2014 (YF18), and genotype VI Pigeon/Guangdong/GZ289/2014 (GZ289), each a predominant chicken-origin, duck-origin, and pigeon-origin genotype, respectively. A total of 27 6-weeks-old SPF white Leghorn chickens were supplied by Guangdong Dahuanong and housed in isolator cages under negative pressure with food and water provided *ad libitum*. A total of 27 pigeons (15-weeks-old *Columba livia* rock pigeons) and 27 commercially available domestic ducks (2-weeks-old Peking ducks) were purchased from a pigeon farm in Gaoming and a duck farm in Yunfu, respectively, and housed in isolators. All pigeons and ducks were confirmed to be serologically negative for ND by HI assays during a 1-day period before experimentation.

### Genetic and phylogenetic analyses

We previously determined the complete genome sequence of the GM strain from LBMs in Guangdong Province preserved in our laboratory (Wang et al., 2014). The complete genome of the other two viruses YF18 and GZ289 used in this study were sequenced. For the other 20 isolates, viral genomic RNA was extracted from infected allantoic fluid by using an RNA isolation kit (RNeasy Mini Kit, Qiagen, Hilden, Germany) according to the manufacturer's instructions and reverse transcribed into cDNA with a cDNA synthesis kit (SuperScript RT-PCR, Invitrogen, Carlsbad, CA, USA). PCR amplification was carried out using primers specific to the complete genomes of NDV, as described previously (Kang et al., 2014), and the NDV F gene open reading frame was amplified using primers, also as described previously (Kang et al., 2014). PCR products were purified with a DNA purification kit (Corning, NY, USA) and sequenced using a cycle sequencing kit (BigDye Terminator, Applied Biosystems, Foster City, CA, USA) according to the manufacturer's protocol. Nucleotide sequences were compiled and edited using Lasergene version 7.1 software (DNASTAR, Madison, WI, USA). A phylogenetic tree based on the F gene open reading frame and the complete genome of the NDVs were constructed by using the maximum likelihood method with the generalized time reversible GTR+G+I4 model, using molecular evolutionary genetics analysis software (MEGA version 5.02), in following (Tamura et al., 2011; Diel et al., 2012). The statistical significance of the tree was assessed with a bootstrap value of 1000.

### Animal experiments

To assess the pathogenicity and transmission of the three representative isolated NDV strains GM, YF18, and GZ289, in chickens, ducks, and pigeons, three groups of 6-weeks-old SPF chickens, 2-weeks-old Peking ducks, and 15-weeks-old *C. livia* rock pigeons (nine birds per group) were inoculated with 10^6^ EID_50_ of the indicated virus in a volume of 200 μL via intraocular and intranasal routes. Eight hour after infection, an additional three chickens, ducks, and pigeons were inoculated intranasally with 200 μL PBS and placed in physical contact—that is, in the same cage and sharing food and water *ad libitum*—with inoculated birds in order to monitor contact infection. We euthanized three inoculated birds (dead or ill with depression, torticollis, incoordination, and tremors) from each subgroup 3 days postinoculated (DPI) to test the virus replication from various tissues from the thymus, cecal tonsils, bursa of Fabricius, trachea, lung, brain, kidney, and spleen. Oropharyngeal and cloacal swabs from all infected and contacted birds were collected for the detection of viruses shedding at 3, 5, 7, 9, and 11 DPI and suspended in 1000 μL transport medium (50% glycerol in PBS) with antibiotics (penicillin, 2000 U/mL; amphotericin B, 2000 U/mL; streptomycin, 2 mg/mL) for viral detection and titration in eggs. Virus titers were calculated as described previously (Kang et al., 2014). All birds were observed daily every 8 h for illness or death during the course of the 14-days experimental period. We collected serum samples from each surviving bird for serologic testing at 14 DPI. All samples were confirmed to show seroconversion by HI test using four HA units of isolates based on standard procedures (OIE, 2012).

### Nucleotide sequence accession numbers

Gen Bank accession numbers of the complete F genes of the 23 South China strains described in this study are designated in Table 1 (KT381585 to KT381606). The complete genome sequences of the GM, YF18, and GZ289 strains obtained in the present study are available from Gen Bank under the accession numbers DQ486859, KR014814, and KR014815, respectively.

## Results

### Pathogenicity analysis

Samples of chickens, ducks, and pigeons were taken at LBMs in Guangzhou, Yunfu, and Gaoming, China, where 42 chickens (*Gallus gallus*), 38 ducks (Peking), and 27 pigeons (*C. livia*) had oropharyngeal and cloacal swab samples taken as part of an avian influenza (H7N9) surveillance program (Table 1). Table 1 presents the initial biological characterizations of 23 NDV isolates, including MDT and ICPI. The seven class I genotype III and four class II genotype I or II samples with MDTs of >120 and with ICPIs of 0–0.2 h were consistent with a lentogenic pathotype. The other 12 strains from South China had MDTs of 42–87 h and ICPIs of 1.22–1.83 and were thus considered velogenic strains (OIE, 2012). Table 1 also lists the F protein cleavage site amino acid sequence of the 23 field isolates of NDV derived in this study, consistent with the virulence pathotype identified by MDT and ICPI tests.

### Genetic and phylogenetic analyses of NDV isolates

**Figure 1:**
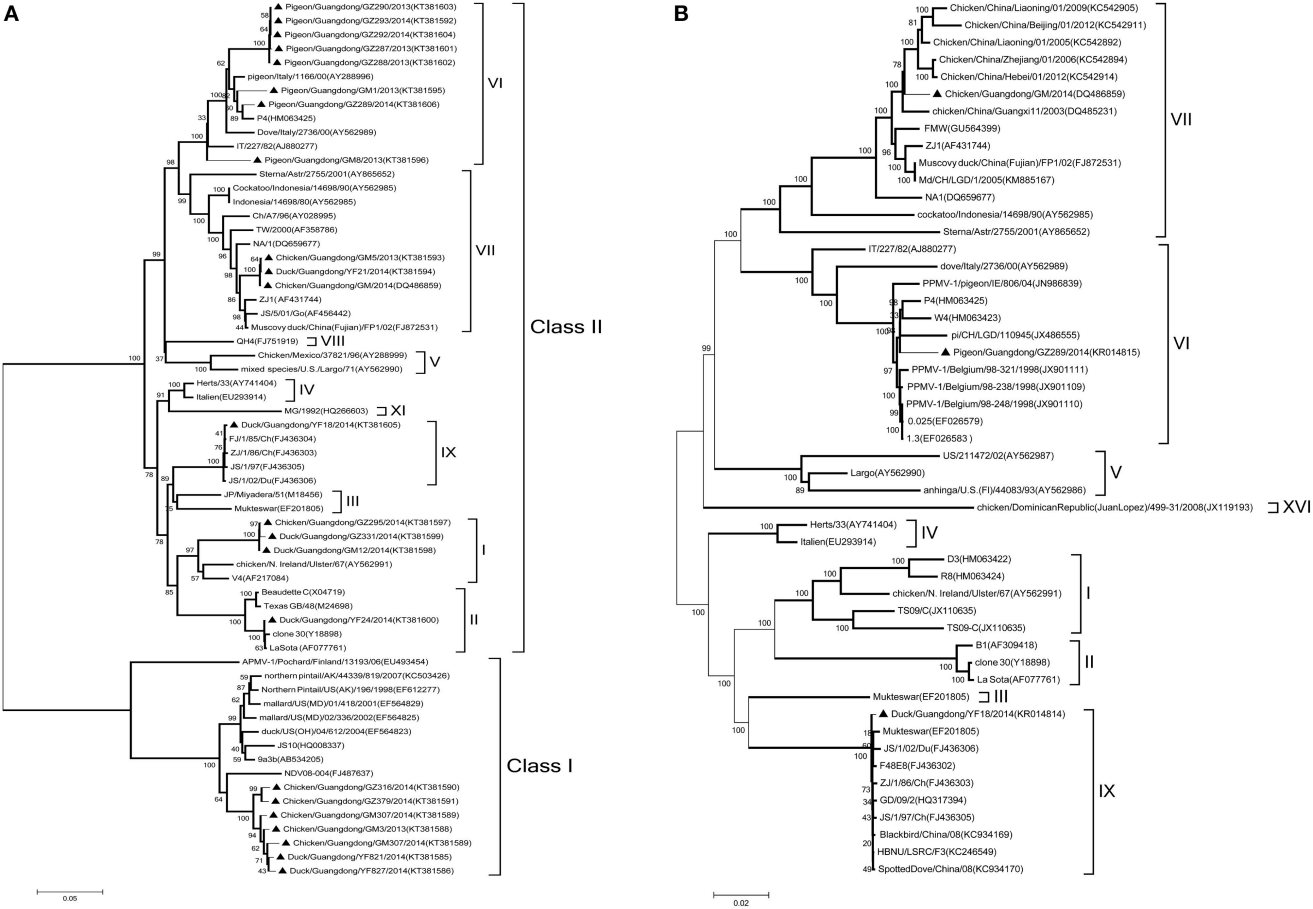
**Phylogenetic tree of NDV strains. (A)** Phylogenetic analysis based on the F-gene open reading frame (1662 nt) of viruses representing NDV class I and II available in Gen Bank. **(B)** Phylogenetic analysis based on the complete genome sequence (15192 nt) of the GM, YF18, and GZ289 strains derived from different birds. Viruses highlighted with the black triangles (▲) were characterized in this study. The tree was constructed using the Maximum Likelihood method with the Generalized Time Reversible GTR+G+I4 model of software MEGA (version 5.02), with 1000 bootstrap trials to assign confidence to the groupings.

Based on the complete F genome sequences derived from different birds (Figure 1A), three strains were assigned to genotypes I, one to genotype II, eight to genotype VI, three to genotype VII, and one to genotype IX, for a total of 16 samples in class II. Seven samples were positive for avirulent class I strains. To further determine their molecular characteristics, we selected three strains noted in the previous section for the complete genome sequenced. The sequences were compared with 52 complete representative NDV genome sequences obtained from Gen Bank. Based on complete nucleotide sequences, phylogenetic analysis revealed that the GM, YF18, and GZ289 strains clustered into class II genotypes VII, IX, and VI, respectively (Figure 1B). In sum, we have shown that 23 NDV strains derived from chickens, ducks, and pigeons in LBMs were virulent or avirulent circulating in South China and have confirmed the coexistence of different genotypes of NDV in domestic poultry *in vivo*, thereby suggesting that NDV may be transmitted between types of domestic poultry and pigeons in South China.

### Pathogenicity and transmission of NDV among SPF chickens

To evaluate tissue tropism and pathogenicity of three representative South China strains GM, YF18, and GZ289, in chickens, we inoculated each chicken with 10^6^ EID_50_ of infective allantoic fluid of the indicated virus in a 200 μL volume via intraocular and intranasal routes and euthanized three chickens from each subgroup at 3 DPI, after which the remaining chickens were observed clinically for 2 weeks.

All chickens exposed to GM died within 3 DPI. All chickens inoculated with YF18 died by 5 DPI. However, none of the chickens infected GZ289 virus died. No symptom was observed in the GZ289-inoculated chickens despite their seroconversion; none of the naïve contact-group chickens seroconverted (Table 2). The GM, YF18, and GZ289 isolates were replicated systemically in various tissues of SPF chickens on 3 DPI, including thymus, cecal tonsils, bursa of Fabricius, trachea, lung, brain, kidney, and spleen (Table 2). The GM, YF18, and GZ289 viruses showed remarkable replication in the lungs; with mean titers were 8.75, 8.25, and 3.42 log_10_EID_50_, respectively. In addition, the selected three viruses furthermore replicated to the mean titer of 6.75, 6.75, 2.00 log_10_EID_50_ in the spleen, respectively. In the GM inoculated chicken, the mean titers were from 6.50 to 8.75 log_10_EID_50_ in the tested tissues, which were greater than those in the YF18 and GZ289-inoculated chickens (Table 2). In other words, the GM virus replicated more highly in the chickens.

**Table 2:** **Lethality, seroconversion, and tissues tropism among chickens in an intraspecies study of NDV transmission^a^**.

**Viruses**	**Manifestations of chickens**				**Virus replication on 3 DPI (log**_**10**_**EID**_**50**_**/g)**^d^ **in:**							
	**No. D/S/total^b^**		**No. S.C./total^c^**									
	**Infected^e^**	**Contact^f^**	**Infected**	**Contact**	**Thymus**	**Cecal tonsils**	**Bursa of Fabricius**	**Trachea**	**Lung**	**Brain**	**Kidney**	**Spleen**
GM	3/3/3	3/3/3	–	–	7.33 ± 0.14	6.92 ± 0.58	6.83 ± 0.14	5.50 ± 0.43	7.75 ± 0.50	6.13 ± 0.53	7.17 ± 0.14	6.75 ± 0.25
YF18	3/3/3	3/3/3	–	–	6.25 ± 0.50	6.83 ± 0.80	6.58 ± 0.58	5.42 ± 0.29	7.25 ± 0.90	5.50 ± 0.90	7.08 ± 0.52	6.75 ± 0.66
GZ289	0/0/3	0/0/3	3/3 (6.0)^g^	0/3	2.92 ± 0.52	2.58 ± 1.01	2.42 ± 0.58	2.33 ± 0.29	3.42 ± 0.14	2.00 ± 0.43	2.17 ± 0.58	2.00 ± 0.25

a *Chickens were inoculated with 10^6^ EID_50_ of the indicated virus in a volume of 200 μL via the intraocular and intranasal routes; Tissues, including thymus, cecal tonsils, bursa of Fabricius, trachea, lung, brain, kidney, and spleen were collected aseptically on three DPI from three chickens when infected with the indicated virus and homogenized for virus titration in eggs*.

b *No. D/S/total shows the number of dead (D) and sick (S) as well as the total number of chickens from the observation period. The chickens that showed clinical signs, such as depression, torticollis, incoordination and tremors, but recovered at the end of the observation were counted as sick animals*.

c *No. S.C./total shows the number of chickens that seroconverted out of the total number of chickens at the end of the observation period. –, all of the chickens died at the end of the observation*.

d *For statistical analysis, a value of 1.5 was assigned if the virus was not detected from the undiluted sample in three SPF embryonated chicken eggs (Kang et al., 2015). Virus titers are expressed as means ± standard deviation in log_10_EID_50_/g of tissue*.

e *Chickens inoculated with virus*.

f *Three uninoculated chickens were co-housed with infected chickens as a contact group 8 h after inoculation*.

g *Average antibody titer of infected chickens (log_2_)*.

GM, YF18, and GZ289 viruses shedding from the inoculated chickens were detected in oropharyngeal and cloacal swabs at 3, 5, 7, 9, and 11 DPI (Table 3). In the infected chickens, the GM virus was recovered from the oropharyngeal (4.79 log_10_EID_50_)on 3 DPI and from the cloacal (5.29 log_10_EID_50_) on 3 DPI (Table 3). The YF18 virus was shed from the oropharynx in inoculated chickens within 5 DPI (2.50–5.25 log_10_EID_50_). But, it only could be shed from the cloaca within 3 DPI (4.92 log_10_EID_50_). GZ289 virus shedding was detected from oropharyngeal and cloacal swabs only at 3 DPI (3.29 and 2.88 log_10_EID_50_, respectively).

**Table 3:** **Virus titers in oraopharyngeal and cloacal swabs from chickens**.

**Viruses**		**Days post-inoculation (log**_**10**_**EID**_**50**_**/0.1 mL)** ± **SD^a^**									
		**3 Days**		**5 Days**		**7 Days**		**9 Days**		**11 Days**	
		**OP**	**CL**	**OP**	**CL**	**OP**	**CL**	**OP**	**CL**	**OP**	**CL**
GM	Infected^b^	4.79 ± 0.60 (6/6)	5.29 ± 0.40 (6/6)	–	–	–	-	–	–	–	–
	Contact^c^	4.13 ± 0.18 (2/3)	3.75 ± 0.43 (3/3)	4.00 (2/3)	3.92 ± 0.14(3/3)	–	–	–	–	–	–
YF18	Infected	5.25 ± 0.27(5/5)	4.92 ± 0.41(5/5)	2.50 (1/1)	ND (0/1)^d^	–	–	–	–	–	–
	Contact	4.00 ± 0.66 (3/3)	4.17 ± 0.14 (3/3)	3.25 ± 0.43(3/3)	3.25 ± 0.71(2/3)	3.00 (1/2)	3.00 (1/2)	–	–	–	–
GZ289	Infected	3.29 ± 0.62 (6/6)	2.88 ± 0.52 (6/6)	ND (0/3)	ND (0/3)	ND (0/3)	ND (0/3)	ND (0/3)	ND (0/3)	ND (0/3)	ND (0/3)
	Contact	ND (0/3)	ND (0/3)	ND (0/3)	ND (0/3)	ND (0/3)	ND (0/3)	ND (0/3)	ND (0/3)	ND (0/3)	ND (0/3)

*OP, oropharyngeal swabs; CL, cloacal swabs; –, all of the chickens died at the end of the observation*.

a *For statistical purposes, a value of 1.5 was assigned if virus was not detected from the undiluted sample in three embryonated hen's eggs (Kang et al., 2015)*.

b *Chickens inoculated with virus*.

c *Naïve contact chickens housed with those inoculated*.

d *No detected*.

To determine whether these three viruses could be horizontally transmitted among chickens, 8 h after infection, three chickens were inoculated with 200 μL PBS via the same routes as a naïve contact group and housed with those inoculated with the GM, YF18 or GZ289 viruses. In naïve contact-group chickens, housed with inoculated GM chickens during the observed time, died within 7 DPI (Table 2). GM virus was recovered from the oropharyngeal swabs (4.00–4.13 log_10_EID_50_) and from the cloacal swabs (3.75–3.92 log_10_EID_50_) at 3 and 5 DPI (Table 3). The naïve contact chickens housed with YF18 died within 7 DPI (Table 2). The virus shedding was detected from oropharyngeal and cloacal swabs at 3, 5, and 7 DPI (3.00–4.00 log_10_EID_50_ and 3.00–4.17 log_10_EID_50_, respectively). The naïve contact chickens housed with GZ289 virus still survived in 14 DPI, but none seroconverted (Table 2). Meanwhile, GZ289 virus was not recovered from oropharyngeal and cloacal swabs during the trial period from contact birds (Table 3).

**Figure 2:**
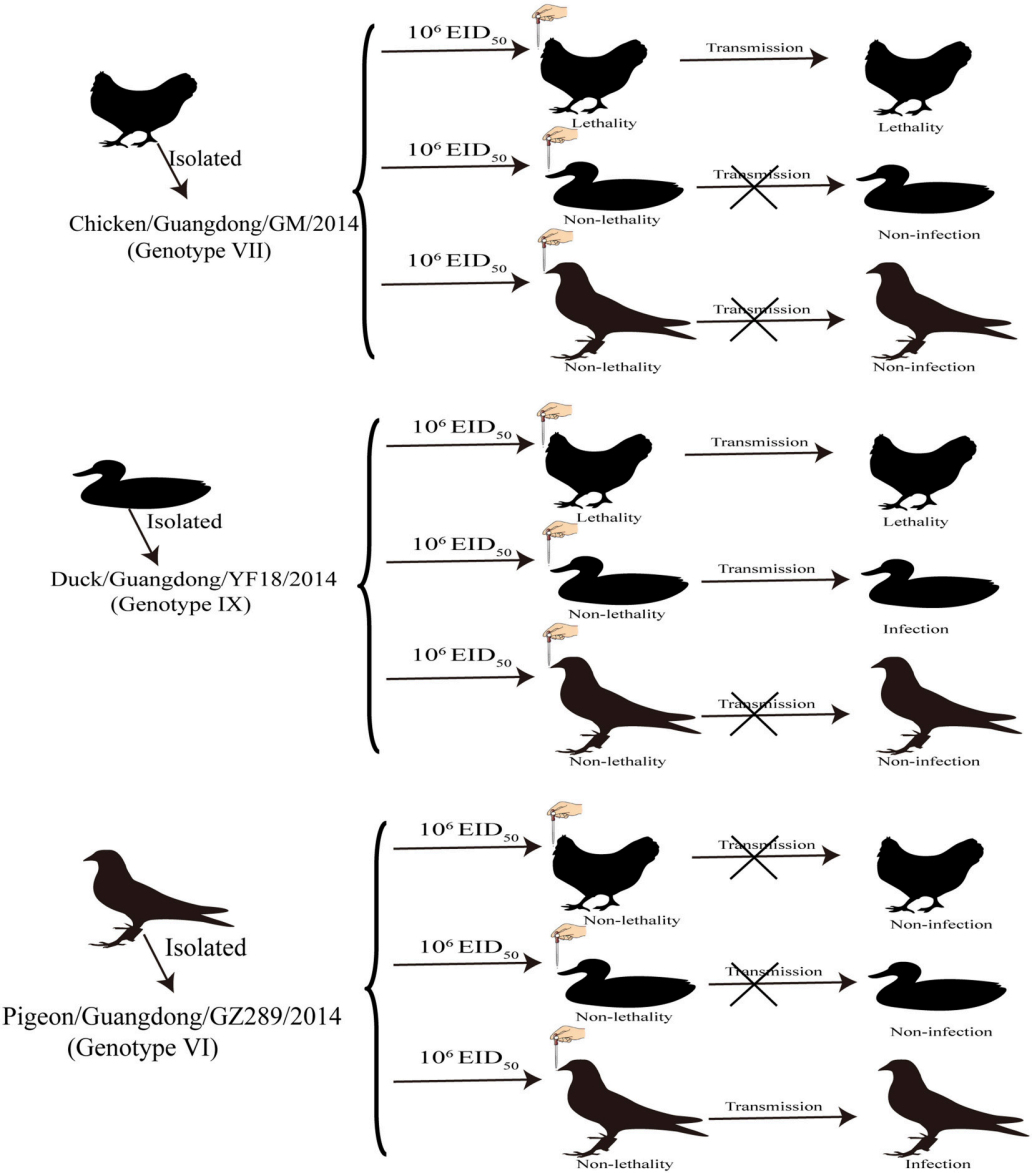
**Transmission studies of NDV in chickens, ducks and pigeons**. Three groups of chickens, ducks, and pigeons were inoculated with 10^6^ EID_50_ of the indicated virus via the intraocular and intranasal routes and, 8 h after infection, three birds were placed into the same cage to monitor contact infection.

Our study indicated that GM and YF18 were highly pathogenic to chickens, and could be transmitted by contact with naïve chickens, while the GZ289 virus did not replicate well in chickens, and did not spread by naïve contact (Figure 2).

### Pathogenicity and transmission of NDV among domestic ducks

To assess the tissue tropism and pathogenicity of the three viruses in ducks, we inoculated each duck with 10^6^ EID_50_ of the indicated virus in 200 μL via intraocular and intranasal routes and euthanized three ducks from each subgroup at 3 DPI. The remaining ducks were observed clinically for 14 days.

All ducks in the three infected virus groups survived during the period of infection (Table 4). The HI titers of groups inoculated with GM, YF18, and GZ289 were 8.0 log_2_, 7.7 log_2_, and 7.3 log_2_, respectively. In the ducks in the contact group, the HI titers for YF18 were all 5 log_2_, though none of the three ducks in the GM and GZ289 groups seroconverted (Table 4).

**Table 4:** **Lethality, seroconversion, and tissues tropism among ducks in an intraspecies study of NDV transmission^a^**.

**Viruses**	**Manifestations of ducks**				**Virus replication on 3 DPI (log**_**10**_**EID**_**50**_**/g)^d^ in:**							
	**No. D/S/total^b^**		**No. S.C./total^c^**									
	**Infected^e^**	**Contact^f^**	**Infected**	**Contact**	**Thymus**	**Cecal tonsils**	**Bursa of Fabricius**	**Trachea**	**Lung**	**Brain**	**Kidney**	**Spleen**
GM	0/0/3	0/0/3	3/3 (8.0)^g^	0/3	3.08 ± 0.29	2.58 ± 0.14	1.67 ± 0.14	ND	4.17 ± 0.14	ND	2.50 ± 0.25	2.42 ± 0.29
YF18	0/0/3	0/0/3	3/3 (7.7)	3/3 (5.0)	3.33 ± 0.14	2.67 ± 0.52	2.75 ± 0.43	1.92 ± 0.29	5.00 ± 0.43	ND	3.58 ± 0.14	2.83 ± 0.38
GZ289	0/0/3	0/0/3	3/3 (7.3)	0/3	2.75 ± 0.43	2.08 ± 0.38	1.58 ± 0.14	ND	3.92 ± 0.63	ND	2.83 ± 0.38	2.58 ± 0.58

a *Ducks were inoculated with 10^6^ EID_50_ of the indicated virus in a volume of 200 μL via the intraocular and intranasal routes; Tissues, including thymus, cecal tonsils, bursa of Fabricius, trachea, lung, brain, kidney, and spleen were collected aseptically on three DPI from three ducks when infected with the indicated virus and homogenized for virus titration in eggs*.

b *No. D/S/total shows the number of dead (D) and sick (S) as well as the total number of ducks from the observation period. The ducks that showed clinical signs, such as depression, torticollis, incoordination, and tremors, but recovered at the end of the observation were counted as sick animals*.

c *No. S.C./total shows the number of ducks that seroconverted out of the total number of ducks at the end of the observation period*.

d *For statistical analysis, a value of 1.5 was assigned if the virus was not detected from the undiluted sample in three SPF embryonated chicken eggs (Kang et al., 2015). Virus titers are expressed as means ± standard deviation in log_10_EID_50_/g of tissue. ND, Not detected*.

e *Ducks inoculated with virus*.

f *Three uninoculated ducks were co-housed with infected ducks as a contact group 8 h after inoculation*.

g *Average antibody titer of infected ducks (log_2_)*.

In the inoculated ducks, the YF18 virus was replicated systemically in the tissues of the thymus, cecal tonsils, bursa of Fabricius, trachea, lung, brain, kidney, and spleen on 3 DPI (Table 4). The YF18 virus replicated more highly in the lungs (5.00 log_10_EID_50_). The mean virus titers in the thymus, cecal tonsils, bursa of Fabricius, trachea, brain, kidney, and spleen were 3.33, 2.67, 2.75, 1.92, 1.58, 3.58, and 2.83 log_10_EID_50_, respectively. Generally, the GM and GZ289 virus titers were lower than those of YF18 in ducks. Those two viruses replicated in some tested tissues, including those of the thymus, cecal tonsils, bursa of Fabricius, lung, kidney, and spleen, but not in those of the trachea or brain (Table 4). The GM and GZ289 viruses replicated more highly in the lungs (4.17 and 3.92 log_10_EID_50_, respectively).

GM, YF18, and GZ289 viruses shedding from the inoculated ducks were detected in oropharyngeal and cloacal swabs at 3, 5, 7, 9, and 11 DPI (Table 5). In the infected ducks, the GM virus could be detected only from oropharyngeal and cloacal swabs at 3 DPI (1.96 and 1.58 log_10_EID_50_, respectively). YF18 virus shedding was detected in the oropharynx in inoculated ducks within 7 DPI, with virus titers from 1.58 to 2.42 log_10_EID_50_, and from the cloaca within 3 DPI, with virus titers of 1.75 log_10_EID_50_. Lastly, GZ289 virus shedding occurred with both oropharyngeal and cloacal swabs at 3 DPI (1.75 and 1.83 log_10_EID_50_, respectively).

**Table 5:** **Virus titers in oropharyngeal and cloacal swabs from ducks**.

**Viruses**		**Days post-inoculation (log**_**10**_**EID**_**50**_**/0.1 mL) ± SD^a^**									
		**3 Days**		**5 Days**		**7 Days**		**9 Days**		**11 Days**	
		**OP**	**CL**	**OP**	**CL**	**OP**	**CL**	**OP**	**CL**	**OP**	**CL**
GM	Infected^b^	1.96 ± 0.71 (2/6)	1.58 ± 0.20 (1/6)	ND^d^(0/3)	ND (0/3)	ND (0/3)	ND (0/3)	ND (0/3)	ND (0/3)	ND (0/3)	ND (0/3)
	Contact^c^	ND(0/3)	ND (0/3)	ND (0/3)	ND (0/3)	ND (0/3)	ND (0/3)	ND (0/3)	ND (0/3)	ND (0/3)	ND (0/3)
YF18	Infected	2.42 ± 1.01 (3/6)	1.75 ± 0.42 (2/6)	1.58 ± 0.14 (1/3)	ND (0/3)	1.58 ± 0.14(1/3)	ND (0/3)	ND (0/3)	ND (0/3)	ND (0/3)	ND (0/3)
	Contact	1.75 ± 0.43 (1/3)	ND (0/3)	ND (0/3)	ND (0/3)	ND (0/3)	ND (0/3)	ND (0/3)	ND (0/3)	ND (0/3)	ND (0/3)
GZ289	Infected	1.75 ± 0.39 (2/6)	1.83 ± 0.38 (3/6)	ND (0/3)	ND (0/3)	ND (0/3)	ND (0/3)	ND (0/3)	ND (0/3)	ND (0/3)	ND (0/3)
	Contact	ND (0/3)	ND (0/3)	ND (0/3)	ND (0/3)	ND (0/3)	ND (0/3)	ND (0/3)	ND (0/3)	ND (0/3)	ND (0/3)

*OP, oropharyngeal swabs; CL, cloacal swabs*.

a *For statistical purposes, a value of 1.5 was assigned if virus was not detected from the undiluted sample in three embryonated hen's eggs (Kang et al., 2015)*.

b *Ducks inoculated with virus*.

c *Naïve contact ducks housed with those inoculated*.

d *No detected*.

To determine whether these viruses could be horizontally transmitted among ducks, 8 h after infection three ducks were inoculated with 200 μL PBS via the same routes as a naïve contact group placed with those inoculated with the GM, YF18, or GZ289 virus. During the experiment period, no ducks in the naïve contact group inoculated with the GM, YF18, or GZ289 virus died (Table 4). In the naïve contact group representing YF18, the virus titers of oropharyngeal swabs were detectable only at 3 DPI (1.75 log_10_EID_50_), whereas cloacal swabs did not show any detectable virus during the period (Table 5). GM and GZ289 virus shedding was not testable in the oropharyngeal or cloacal swabs of the naïve contact duck, even at 14 DPI (Table 5).

In our study of the ducks, the YF18 virus was found to infect ducks and be transmitted among ducks via naïve contact. Although the GM and GZ289 viruses could infect ducks, they could not be transmitted among them by naïve contact (Figure 2).

### Pathogenicity and transmission of NDV among pigeons

To investigate the tissue tropism and pathogenicity of the three viruses in pigeons, we inoculated each pigeon with 10^6^ EID_50_ of the indicated virus in 200 μL via intraocular and intranasal routes and euthanized three pigeons from each subgroup at 3 DPI. All remaining pigeons were observed clinically for 14 days.

No pigeons died during the observation period. In pigeons inoculated with the GZ289 virus, or in those in naïve contact with GZ289 virus-inoculated pigeons, HI titers were far higher than those observed for the two other viruses. In GZ289-inoculated pigeons, three seroconverted and showed high titers (9.3 log_2_), whereas two pigeons in the contact group seroconverted with relatively high titers (6.0 log_2_). The HI titers of groups inoculated with the GM and YF18 virus were 8.3 log2, and 8.7 log_2_, respectively, although none of the three contact pigeons seroconverted (Table 6).

**Table 6:** **Lethality, seroconversion, and tissues tropism among pigeons in an intraspecies study of NDV transmission^a^**.

**Viruses**	**Manifestations of pigeons**				**Virus replication on 3 DPI (log**_**10**_**EID**_**50**_**/g)^d^ in:**							
	**No. D/S/total^b^**		**No. S.C./total^c^**									
	**Infected^e^**	**Contact^f^**	**Infected**	**Contact**	**Thymus**	**Cecal tonsils**	**Bursa of Fabricius**	**Trachea**	**Lung**	**Brain**	**Kidney**	**Spleen**
GM	0/0/3	0/0/3	3/3 (8.3)^g^	0/3	3.08 ± 0.76	3.92 ± 0.52	2.33 ± 0.63	1.83 ± 0.58	4.42 ± 0.14	2.42 ± 0.29	3.08 ± 0.29	2.92 ± 0.29
YF18	0/0/3	0/0/3	3/3 (8.7)	0/3	1.67 ± 0.14	1.83 ± 0.38	ND	ND	2.33 ± 0.52	ND	1.75 ± 0.43	2.08 ± 0.52
GZ289	0/1/3	0/0/3	3/3 (9.3)	2/3 (6.0)	3.83 ± 0.58	4.33 ± 0.38	4.58 ± 0.14	2.83 ± 0.38	5.33 ± 0.38	2.75 ± 0.43	3.75	4.92 ± 1.01

a *Pigeons were inoculated with 10^6^ EID_50_ of the indicated virus in a volume of 200 μL via the intraocular and intranasal routes; Tissues, including thymus, cecal tonsils, bursa of Fabricius, trachea, lung, brain, kidney, and spleen were collected aseptically on three DPI from three pigeons when infected with the indicated virus and homogenized for virus titration in eggs*.

b *No. D/S/total shows the number of dead (D) and sick (S) as well as the total number of pigeons from the observation period. The pigeons that showed clinical signs, such as depression, torticollis, incoordination, and tremors, but recovered at the end of the observation were counted as sick animals*.

c *No. S.C./total shows the number of pigeons that seroconverted out of the total number of pigeons at the end of the observation period*.

d *For statistical analysis, a value of 1.5 was assigned if the virus was not detected from the undiluted sample in three SPF embryonated chicken eggs (Kang et al., 2015). Virus titers are expressed as means ± standard deviation in log_10_EID_50_/g of tissue. ND, Not detected*.

e *Pigeons inoculated with virus*.

f *Three uninoculated pigeons were co-housed with infected pigeons as a contact group 8 h after inoculation*.

g *Average antibody titer of infected pigeons (log_2_)*.

The GM and GZ289 viruses replicated systemically in pigeons, which was detectable in all tested tissues at 3 DPI, including those of the thymus, cecal tonsils, bursa of Fabricius, trachea, lung, brain, kidney, and spleen (Table 6). The YF18 virus replicated only in some tested tissues, including those of the thymus, lung, cecal tonsils, kidney, and spleen; mean virus titers were 1.67, 2.33, 1.83, 1.75, and 2.08 log_10_EID_50_, respectively. The GM, YF18, and GZ289 viruses showed remarkable replication in the lungs, with mean titers of 4.42, 2.33, and 5.33 log_10_EID_50_, respectively. The three selected viruses furthermore replicated in the spleen to mean titers of 2.92, 2.08, and 4.92 log_10_EID_50_. These results indicate that GZ289 replicated more highly than the other two viruses in tested tissues of infected pigeons.

GM, YF18, and GZ289 viruses shedding from the inoculated pigeons were detected in oropharyngeal and cloacal swabs at 3, 5, 7, 9, and 11 DPI (Table 7). The GM virus could be isolated from both the oropharyngeal and cloacal swabs within 5 DPI (1.54–1.83 and 1.83–2.42 log_10_EID_50_, respectively). In the YF18 virus- inoculated group, the virus titers of the cloacal swabs were detectable only at 3 DPI (1.75 log_10_EID_50_); however, the virus titers of the oropharyngeal swabs could not be detected during our observation period (Table 7). The GZ289 virus was shed from both the oropharyngeal and cloacal swabs in inoculated pigeons within 11 DPI, except at 3 DPI (1.58–3.83 and 1.92–3.33 log_10_EID_50_, respectively).

**Table 7:** **Virus titers in oropharyngeal and cloacal swabs from pigeons**.

**Viruses**		**Days post-inoculation (log**_**10**_**EID**_**50**_**/0.1 mL) ± SD^a^**									
		**3 Days**		**5 Days**		**7 Days**		**9 Days**		**11 Days**	
		**OP**	**CL**	**OP**	**CL**	**OP**	**CL**	**OP**	**CL**	**OP**	**CL**
GM	Infected^b^	1.54 ± 0.10(1/6)	1.83 ± 0.52(2/6)	1.83 ± 0.38(2/3)	2.42 ± 1.01(2/3)	ND^d^ (0/3)	ND (0/3)	ND (0/3)	ND (0/3)	ND (0/3)	ND (0/3)
	Contact^c^	ND (0/3)	ND (0/3)	ND (0/3)	ND(0/3)	ND (0/3)	ND (0/3)	ND (0/3)	ND (0/3)	ND (0/3)	ND (0/3)
YF18	Infected	ND (0/3)	1.75 ± 0.42(2/6)	ND (0/3)	ND(0/3)	ND (0/3)	ND (0/3)	ND (0/3)	ND (0/3)	ND (0/3)	ND (0/3)
	Contact	ND (0/3)	ND (0/3)	ND (0/3)	ND(0/3)	ND (0/3)	ND (0/3)	ND (0/3)	ND (0/3)	ND (0/3)	ND (0/3)
GZ289	Infected	ND (0/3)	ND (0/3)	2.00 ± 0.50(2/3)	2.83 ± 0.95(2/3)	3.83 ± 1.15(3/3)	3.33 ± 0.59(3/3)	2.79 ± 0.99(3/3)	2.71 ± 0.86(3/3)	1.58 ± 0.14(1/3)	1.92 ± 0.29(2/3)
	Contact	ND (0/3)	ND (0/3)	1.75 ± 0.43(2/3)	ND(0/3)	1.83 ± 0.58(2/3)	1.67 ± 0.14(2/3)	1.67 ± 0.29(1/3)	1.58 ± 0.14(1/3)	ND (0/3)	ND (0/3)

*OP, oropharyngeal swabs; CL, cloacal swabs*.

a *For statistical purposes, a value of 1.5 was assigned if virus was not detected from the undiluted sample in three embryonated hen's eggs (Kang et al., 2015)*.

b *Pigeons inoculated with virus*.

c *Naïve contact pigeons housed with those inoculated*.

d *No detected*.

To determine whether these three viruses could be horizontally transmitted among pigeons, 8 h after infection, three pigeons were inoculated with 200 μL PBS via the same routes as a naïve contact group placed with those inoculated with the GM, YF18, or GZ289 viruses. No pigeons died in the naïve contact subgroup placed with those exposed to the three selected viruses (Table 6). No virus in the naïve contact pigeons with GM or YF18 could be isolated from oropharyngeal or cloacal swabs (Table 7). In the naïve contact group with GZ289, virus shedding could be detected from oropharyngeal swabs at 5, 7, and 9 DPI (1.67–1.83 log_10_EID_50_) and tested from cloacal swabs only at 7 and 9 DPI (1.58–1.67 log_10_EID_50_).

In sum, the GZ289 virus could infect and transmit among pigeons by naïve contact. Though the GM and YF18 viruses could infect pigeons, they could not transmit among pigeons by naïve contact (Figure 2).

## Discussion

South China's Guangdong Province is considered to be an ideal transmission area for NDV. It hosts numerous large-scale LBMs and live poultry markets, as well as a multitude of small backyard farms and small-scale poultry farms (Shortridge and Stuart-Harris, 1982). These poultry, including chickens, ducks, geese, pigeons, and numerous other species, are traded in LBMs daily. Due to the constant close proximity of these poultry, viruses achieve transmission in different birds and contribute to emergent novel NDVs. Indeed, South China is considered to be a virus epicenter, due to large-scale severe acute respiratory syndrome, high pathogenic avian influenza, H5N1, and dengue outbreaks (Shortridge and Stuart-Harris, 1982; Qiu et al., 1993; Zhong et al., 2003; Chen et al., 2004). We therefore propose conducting routine surveillance, using chilled instead of live poultry for sale, and temporary rest days in poultry markets to prevent the intra- and interspecies transmission of NDV.

In recent years, NDV has caused large-scale outbreaks in poultry in many countries around the world, including China (Zhang et al., 2011; Chong et al., 2013; Kang et al., 2014), Japan (Mase et al., 2002), Southern Brazil (Marks et al., 2014), Indonesia (Xiao et al., 2012), South America (Diel et al., 2012), and West Malaysia (Jaganathan et al., 2015). In China, though the implementation of intensive vaccination and the culling of infected birds are effective policies for controlling ND in poultry and rural farms, virulent NDV can still frequently be isolated in vaccinated poultry (Liu et al., 2003; Zhang et al., 2011; Kang et al., 2014). Genetic and phylogenetic studies have shown that NDV is continuously evolving, with viruses of different genotypes undergoing simultaneous changes in distinct geographic areas (Diel et al., 2012; Chong et al., 2013). In our study, we characterized genetic and pathotypic properties of NDV strains isolated from chickens, ducks, and pigeons in LBMs in the province. The genetic and phylogenetic analysis of the complete sequences of the F protein gene showed that seven of 23 poultry-derived strains were avirulent class I NDV, three of 23 were class II genotype I, and one strain was class II genotype II. These results indicate that, similar to low pathogenic avian influenza, lentogenic NDVs prevalently circulate among domestic poultry at LBMs (Seal et al., 2005; Zhu et al., 2014). Additionally, as results of phylogenetic analyses show, 12 velogenic strains isolated from different birds related to predominant strains of class II genotypes VI, VII, and IX, thus suggesting the coexistence of different genotypes of NDV circulating simultaneously in South China, as well as a high probability of the emergence of new strains via recombination. Accordingly, epidemiological surveillance and further investigation at LBMs in South China is necessary in order to clarify the genetic evolution of NDV and thus issue early warnings.

PPMV-1 is generally virulent, though upon infecting chickens can result in clinical diseases expected of NDV with low virulence (OIE, 2012); however, these viruses remain a hidden threat to the poultry industry (de Oliveira Torres Carrasco et al., 2008). Previous studies have demonstrated that PPMV-1 strains are capable of being transmitted from infected pigeons to chickens and turkeys housed in physical contact, as well as that systemic replication can occur in those chickens and turkeys, as shown by the shedding the virus via oropharyngeal and cloacal routes and a humoral immune response to the virus; however quails and geese did not exhibit any clinical signs or shed the virus (Alexander and Parsons, 1984; Smietanka et al., 2014). Very few studies have examined the infectivity, pathogenesis, and transmission of NDV and PPMV-1 infections in different birds. In response, the aim of our study was to investigate the susceptibility and transmission of chickens, ducks, and pigeons following infection with three NDV strains—namely, Chicken/Guangdong/GM/2014 (GM), Duck/Guangdong/YF18/2014 (YF18), and Pigeon/Guangdong/GZ289/2014 (GZ289)—and to provide useful information for improving control strategies against ND. Our results demonstrate that GM is highly pathogenic to chickens and can transmit among them as well as ducks while circulating in chickens. YF18 was highly pathogenic in chickens, might have moderate or low pathogenicity in ducks and pigeons, and does not transmit to pigeons. In addition, GZ289 isolated from pigeons showed low pathogenicity to chickens and domestic ducks and could transmit only in pigeons (Figure 2). These results showed that NDVs isolated from different birds exhibit different host ranges and tissue tropisms. Nevertheless, our study posed several limitations—for instance, we do not know the infective dose for each virus for each species, owing to the adaptability of a virus within a single species.

At least one previous study has reported that pigeons exhibited high morbidity and mortality rates, whereas chickens showed no clinical signs, when infected with the same PPMV-1 strain (Guo et al., 2014). However, opposite results were found by Dortmans et al. (2011)—ones consistent with the results of our experiments—who failed to induce clinical signs in pigeons infected with pigeon strain AV324 or FL-Herts, though the virus was shed from the oropharynx and cloaca in inoculated pigeons (Dortmans et al., 2011). These findings suggest that the course of experimental infection with PPMV-1 in different birds can vary greatly and most likely depends on the infective dose for each of the viruses, the inoculation route, the immunity of the host, and the age and species of the birds.

Current NDV vaccines in circulation, including class II genotype II vaccine virus (B1, Clone-30, and La Sota) and genotype I vaccine virus (V4), are still used at a large scale, most extensively for protecting poultry flocks from ND in South China (Hu et al., 2009). However, until now, well-controlled findings have not demonstrated the role of vaccination in any attempt to control NDV outbreaks by preventing virus transmission in poultry flocks. Moreover, current vaccines can prevent NDV outbreaks, yet not stop viral shedding in vaccinated poultry flocks (Kapczynski and King, 2005). Additional studies are therefore needed to identify the best vaccine candidate, not only for preventing clinical disease and mortality, but also to decrease the magnitude of viral shedding from vaccinated birds.

A correlation exists between antibody response and shedding after infection with virulent NDV in susceptible animals (Miller et al., 2013). During the course of our study, the statistical analysis of serological results showed significant differences among chicken, duck, and pigeon groups exposed to different viruses, as well as the naïve contacts. In chickens in the contact group, the virus was detectable from oropharyngeal and cloacal swabs inoculated with GM and YF18, whereas in ducks in the contact group, we could detect the virus only from oropharyngeal and cloacal swabs inoculated with YF18, with HI titers for the GM, YF18, and GZ289 of 5, 6, and 4 log_2_, respectively. In contact group pigeons, the virus could be detected only from oropharyngeal and cloacal swabs inoculated with GZ289. Moreover, HI titers for GZ289 at 14 DPI were all 6 log_2_, though none of the three pigeons in the GM and YF18 groups seroconverted (HI titers = 4). In all, the efficient replication, high seroconversion, and shedding of relatively high titers in naïve contact groups suggest that NDV isolated from different birds was transmitted to the naïve contact group.

Altogether, our results provide clear evidence that genetically diverse viruses circulate in LBMs in South China's Guangdong Province and illustrate that the three NDV strains isolated from different birds have varying levels of infectivity, pathogenicity, and transmission in chickens, ducks, and pigeons. Our findings thus emphasize the need for constant epidemiological studies in LBMs, in order to enhance active surveillance toward preventing the spread and evolution of these viruses.

## Author contributions

YK conceived the study and wrote the paper. BX and YK designed, performed, and analyzed all the experiments. RY provided technical assistance and prepared all the figures. YL, SF, YL, and TR designed the study and revised the manuscript. All authors reviewed the results and approved the final version of the manuscript.

### Conflict of interest statement

The authors declare that the research was conducted in the absence of any commercial or financial relationships that could be construed as a potential conflict of interest.
